# Insular activation and functional connectivity in firefighters with post-traumatic stress disorder

**DOI:** 10.1192/bjo.2022.32

**Published:** 2022-03-15

**Authors:** Deokjong Lee, Jung Eun Lee, Junghan Lee, Changsoo Kim, Young-Chul Jung

**Affiliations:** Department of Psychiatry, Yongin Severance Hospital, Yonsei University College of Medicine, Yongin, South Korea; and Institute of Behavioral Science in Medicine, Yonsei University College of Medicine, Seoul, South Korea; Department of Psychiatry, Seongnam Saran Hospital, Seongnam, South Korea; Department of Psychiatry, Severance Hospital, Yonsei University College of Medicine, Seoul, South Korea; and Institute of Behavioral Science in Medicine, Yonsei University College of Medicine, Seoul, South Korea; Department of Preventive Medicine, Yonsei University College of Medicine, Seoul, South Korea; and Department of Public Health, Yonsei University Graduate School, Seoul, South Korea; Department of Psychiatry, Severance Hospital, Yonsei University College of Medicine, Seoul, South Korea; and Institute of Behavioral Science in Medicine, Yonsei University College of Medicine, Seoul, South Korea

**Keywords:** Post-traumatic stress disorder, functional MRI, insula, firefighters

## Abstract

**Background:**

Firefighters are frequently exposed to stressful situations and are at high risk of developing post-traumatic stress disorder (PTSD). Hyperresponsiveness to threatening and emotional stimuli and diminishment of executive control have been suggested as manifestations of PTSD.

**Aims:**

To examine brain activation in firefighters with PTSD by conducting an executive control-related behavioural task with trauma-related interferences.

**Method:**

Twelve firefighters with PTSD and 14 healthy firefighters underwent functional magnetic resonance imaging (fMRI) while performing a Stroop match-to-sample task using trauma-related photographic stimuli. Seed-based functional connectivity analysis was conducted using regions identified in fMRI contrast analysis.

**Results:**

Compared with the controls, the participants with PTSD had longer reaction times when the trauma-related interferences were presented. They showed significantly stronger brain activation to interfering trauma-related stimuli in the left insula, and had weaker insular functional connectivity in the supplementary motor area and the anterior cingulate cortex than the controls. They also showed a significant correlation between left insula–supplementary motor area connectivity strength and the hyperarousal subscale of the Clinician-Administered PTSD Scale.

**Conclusions:**

Our findings indicate that trauma-related stimuli elicit excessive brain activation in the left insula among firefighters with PTSD. Firefighters with PTSD also appear to have weak left insular functional connectivity with executive control-related brain regions. This aberrant insular activation and functional connectivity could be related to the development and maintenance of PTSD symptoms in firefighters.

Post-traumatic stress disorder (PTSD) presents with a variety of psychiatric symptoms, including intrusion, avoidance and hyperarousal, which arise after experiencing one or multiple traumatic events. The psychiatric symptoms of PTSD harm several domains of cognitive functioning; for example, the negative impact of PTSD on attentional and executive functioning is well established.^1^ Impaired executive function is further associated with both the development and maintenance of PTSD and its clinical manifestations.^2^ Interestingly, research has indicated that difficulty with executive function in people with PTSD is more prominent in trauma-related contexts^3^ and that individuals with PTSD have difficulty exercising top-down executive control while engaging with stress-related stimuli.^4^

Functional brain imaging studies have identified functional abnormalities in several areas of the brains of people with PTSD. Functional magnetic resonance imaging (fMRI) studies of PTSD have revealed reduced functional activation of the anterior cingulate cortex (ACC) and medial prefrontal cortex (MPFC), both of which are related to top-down executive control,^5,6^ as well as increased functional activation of the amygdala and the insula, reflecting hyperresponsiveness to stimuli.^7,8^ These findings are particularly associated with hyperresponsivity when assessing the salience of emotional or threating stimuli. Although fMRI studies have provided important evidence for clarifying the neurobiological mechanisms of PTSD, their results are complex, inconsistent and dependent on the design of the experiments (e.g. which people participated, which tasks were undertaken and which stimuli participants were exposed to).

Firefighters are often faced with stressful situations, including threatening and traumatic events. This psychosocial work environment exposes firefighters to stressors associated with the development of PTSD.^9^ Research has shown that firefighters experience PTSD symptoms more often than the general population^10^ and that the physical and psychological stressors they face in their work environments have a negative impact on their cognitive functioning.^11^ Given that PTSD affects executive control – an important domain of cognitive function and a crucial resilience factor in stress reactions^12^ – assessing executive control in firefighters with PTSD and the brain activation associated with it is important in identifying the mechanisms of PTSD.

## Aims

The purpose of this study was to identify the pathophysiology of PTSD by analysing functional brain activation patterns in firefighters with PTSD. We applied a Stroop match-to-sample task to evaluate the functional activation related to executive control in these firefighters. Photographic stimuli related to the participants’ occupational environment were inserted into the Stroop task as trauma-related interference while they conducted the task. To investigate functional brain activation related to the trauma-related interference stimuli, we conducted functional brain contrast analysis in firefighters with PTSD and compared the results with those of firefighters without PTSD.

## Method

### Participants

Firefighters who had not received psychiatric treatment but complained of PTSD-related symptoms were recruited for voluntary participation. This study's participants were previously included in the Firefighter Research on the Enhancement of Safety and Health (FRESH) cohort^13^ and had therefore been assessed for their physical and psychological health status. All participants completed the Korean version of the Posttraumatic Diagnostic Scale (PDS) for PTSD screening.^14^ A board-certified psychiatrist evaluated all participants using the Structured Clinical Interview for DSM-IV Axis I Disorders (SCID-I) and confirmed whether each participant met the criteria for PTSD diagnosis.^15^ Individuals who received a PDS score of 15 or higher and met the PTSD diagnostic criteria in the clinical interview were assigned to the PTSD group; 1 male participant had a high PDS score but was excluded because he did not meet the diagnostic criteria. Participants who scored less than 15 points on the PDS and did not meet the PTSD criteria in the clinical interview were classified as controls. Afterwards, the participants’ PTSD-related features were assessed according to the Clinician-Administered PTSD Scale (CAPS) through clinical interviews conducted by a psychiatrist.^16^ The presence of comorbid psychiatric disorders was assessed via the SCID-I.^15^ Participants were excluded if they had a current or past non-PTSD psychiatric illness, history of psychiatric medication use, traumatic brain injury, neurological disease, visual defect or contraindication for MRI. One female was excluded owing to her history of major depressive disorder. This study's participants ultimately included 26 right-handed men and women (control group, *n* = 14; PTSD group, *n* = 12). The groups each included one woman; the rest were men. All participants completed a series of questionnaires after PTSD screening, including the Center for Epidemiologic Studies Depression scale (CES-D),^17^ the Beck Anxiety Inventory (BAI),^18^ the Alcohol Use Disorders Identification Test (AUDIT)^19^ and the Pittsburgh Sleep Quality Index (PSQI).^20^ Each participant's full-scale IQ was measured using the Wechsler Adult Intelligence Scale – Fourth Edition (WAIS-IV).^21^

### Ethics statement

The authors assert that all procedures contributing to this work comply with the ethical standards of the relevant national and institutional committees on human experimentation and with the Helsinki Declaration of 1975, as revised in 2008. All procedures involving human patients were approved by the Ethics Committee of Severance Hospital, Yonsei University Health System in Seoul, Korea (approval no. 4-2016-0187). Written informed consent was obtained after a full description of the scope of the study was given to all participants.

### Stroop match-to-sample task using trauma-related stimuli

**Fig. 1:**
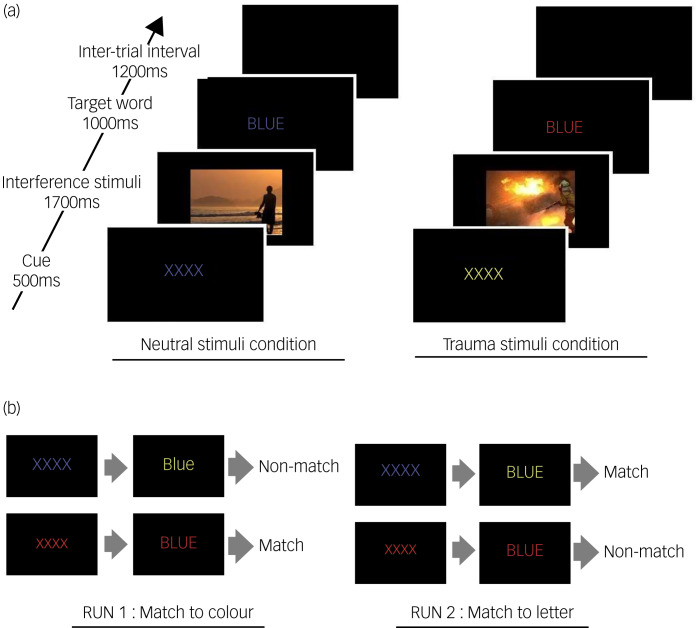
Stroop match-to-sample task. (a) Examples for trauma-related and neutral stimuli presentations. (b) Examples of the experimental paradigm.

We used the Stroop match-to-sample task to explore the brain's response to executive control performance when interfering stimuli were introduced,^22^ following the same task structure as was used in our previous study of eating disorders (Fig. 1).^23^ The detailed conditions of the task, such as the compositions of Run 1 and Run 2 and the length of time the stimulus was presented, were all identical to those of the previous study. In Run 1, participants responded according to the written colour of a target word; in Run 2, participants responded according to the colour that a target word referred to. Accuracy and reaction time were measured for each participant. However, unlike our previous study, which used photographs of food, this study compared the ‘trauma-related condition’ with the ‘neutral condition’ using trauma-related and neutral stimuli respectively. The trauma-related stimuli consisted of photographs of firefighting or emergency rescues, whereas the neutral stimuli consisted of photographs of daily life. The photographs were selected from 55 trauma-related and 55 daily life-related photographs through a preliminary questionnaire survey. The six trauma-related photos with the most negative valence and highest tension were selected as the trauma-related stimuli, and the six neutral photos with the most positive valence and lowest tension were selected as the neutral stimuli.

### Image acquisition and pre-processing

MRI data were acquired using a 3 T Siemens Magnetom MRI scanner (Siemens AG, Erlangen, Germany) equipped with an 8-channel head coil. The *T*_2_-weighted gradient echo-planar pulse sequence was as follows: echo time TE = 30 ms, repetition time TR = 2200 ms, flip angle 90°, field of view 240 mm, matrix 64 × 64, slice thickness 4 mm. The *T*_1_-weighted spoiled gradient echo sequence was as follows: TE = 2.19 ms, TR = 1780 ms, flip angle 9°, field of view 256 mm, matrix 256 × 256, slice thickness 1 mm. Spatial pre-processing and statistical analyses of the functional images were conducted using the Statistical Parametric Mapping 12 software for Windows (SPM12; Wellcome Centre for Human Neuroimaging). To ensure that there was no sudden head movement and that the maximum head movement in each axis was <3 mm, estimates of the realignment parameters for each participant were visually examined. Functional images for each participant were realigned and registered to structural images, which were segmented according to grey matter, white matter and cerebrospinal fluid. The images were then normalised into standard Montreal Neurological Institute (MNI) space. Spatial parameters were entered into the realigned and unwrapped functional images to re-sample them to a 2 mm isotropic voxel size. Smoothing was applied using an 8 mm full-width at half-maximum kernel.

### fMRI contrast analysis and functional connectivity analysis

Individual statistics were computed using a general linear model approach in SPM12. In the first-level analysis, blood oxygen level-dependent (BOLD) contrast images for the ‘trauma-related condition’ blocks and ‘neutral condition’ blocks were generated for each participant. The resulting set of contrast images was then applied into a second-level analysis using a full-factorial model. Two-way analysis of variance (ANOVA) was conducted for brain voxels with the group (PTSD group and control group) as the between-group factor and the task condition (trauma-related and neutral conditions) as the within-group factor to compare the functional connectivity estimates between the PTSD and control groups. Several brain regions were set as regions of interest (ROIs) to conduct the fMRI contrast analysis: both sides of the MPFC, the ACC, the amygdala and the insula. These regions were delineated by the Automated Anatomical Labeling atlas^24^ provided in the Wake Forest University PickAtlas toolbox.^25^ Initially, a voxel-wise cluster-defining threshold of uncorrected *P* < 0.001 was applied. Then, we reported significant clusters with a cluster-level extent threshold correction of false-wise error rate of *P* < 0.05.

Seed-based functional connectivity analysis was performed using the regions identified in the fMRI contrast analysis. The CONN-fMRI functional connectivity toolbox (www.nitrc.org/projects/conn) was used to create individual seed-to-voxel functional connectivity maps. The waveform of each brain voxel was filtered using a bandpass filter (0.008 Hz < *f* < 0.09 Hz) to minimise the effect of low-frequency drift and high-frequency noise. Signals from ventricular regions and white matter were eliminated from the data using linear regression. Correlation coefficients were extracted and converted to *z*-values to estimate functional connectivity strengths using Fisher's *r*-to-*z* transformation. Then, the functional connectivity strength estimates at each voxel were compared between groups using ANOVA. Statistical inferences for the whole-brain analyses were set to a voxel-wise cluster-defining threshold of uncorrected *P* < 0.001, with a cluster-level extent threshold correction of false-wise error rate of *P* < 0.05.

### Statistical analysis

Statistical analyses were performed using SPSS version 24.0 statistical software for Windows (SPSS, Chicago IL, USA) and the threshold for statistical significance was set at *P* < 0.05. Demographic, clinical and behavioural variables were compared using a two-sample *t*-test. We conducted correlation analyses to examine the relationship between brain functional characteristics and clinical features. The functional connectivity strength was calculated by extracting the Fisher-transformed *z*-values of functional connectivity between the left insula seed and the target regions that were identified through functional connectivity analysis. Age- and BAI-controlled partial correlation analyses were conducted to determine whether the functional connectivity estimates correlated with the scores on the CAPS subscales and/or behavioural variables of the Stroop match-to-sample task.

## Results

### Clinical characteristics

There were no statistically significant differences between the PTSD and control groups in age, gender or IQ (Table 1). The PTSD group had higher PDS and CAPS scores than the control group (PDS: *P* < 0.0001; CAPS: *P* < 0.001). The PTSD group had higher scores than the control group on all subscales of the CAPS (intrusion: *P* < 0.001; avoidance: *P* < 0.001; hyperarousal: *P* < 0.001). There were no statistically significant differences between the PTSD and control groups in the CES-D, AUDIT and PSQI results. The PTSD group had higher BAI scores than the controls (*P* = 0.010). The comparison of behavioural performance on the Stroop match-to-sample task showed a difference only in reaction times in the trauma-related condition (*P* = 0.044; Table 2).

**Table 1:** Demographic and clinical variables of participants

	PTSD group (*n* = 12), mean (s.d.)	Control group (*n* = 14), mean (s.d.)	Test	*P*
Age, years	52.2 (8.1)	44.6 (11.1)	*t* = 1.950	0.063
Gender (male)	11 (91.7)^a^	13 (92.9)^a^	χ^2^ = 0.013	0.910
Full-scale IQ score	108.3 (9.2)	114.2 (8.6)	*t* = −1.707	0.101
PDS score	21.3 (4.8)	8.5 (5.1)	*t* = 6.711	<0.001
CAPS total score	56.8 (11.3)	15.1 (10.2)	*t* = 9.880	<0.001
Intrusion	16.8 (3.3)	3.3 (2.8)	*t* = 11.259	<0.001
Avoidance	25.8 (6.4)	9.4 (6.6)	*t* = 6.431	<0.001
Hyperarousal	14.2 (5.4)	2.4 (2.6)	*t* = 6.970	<0.001
CES-D score	14.3 (8.8)	9.1 (8.9)	*t* = 1.487	0.150
BAI score	17.0 (9.9)	6.9 (8.7)	*t* = 2.780	0.010
AUDIT score	10.8 (8.8)	10.4 (8.8)	*t* = 0.114	0.911
PSQI score	7.7 (3.0)	7.5 (3.3)	*t* = 0.134	0.895

AUDIT, Alcohol Use Disorders Identification Test; BAI, Beck Anxiety Inventory; CAPS, Clinician-Administered Post-Traumatic Stress Disorder Scale; CES-D, Center for Epidemiologic Studies Depression scale; PDS, Posttraumatic Diagnostic Scale; PSQI, Pittsburgh Sleep Quality Index; PTSD, post-traumatic stress disorder.

a. Data on gender are expressed as *n* (%).

**Table 2:** Behavioural performance results

	PTSD group (*n* = 12), mean (s.d.)	Control group (*n* = 14), mean (s.d.)	Test	*P*
Accuracy, %				
Trauma-related stimuli	81.8 (15.4)	90.0 (7.2)	*t* = −1.704	0.109
Neutral stimuli	83.5 (15.5)	90.9 (8.4)	*t* = −1.482	0.157
Reaction time, ms				
Trauma-related stimuli	679.6 (89.8)	603.7 (91.4)	*t* = 2.129	0.044
Neutral stimuli	662.3 (89.2)	592.7 (91.5)	*t* = 1.957	0.062

PTSD, post-traumatic stress disorder.

### Brain activation associated with interfering trauma-related stimuli

**Fig. 2:**
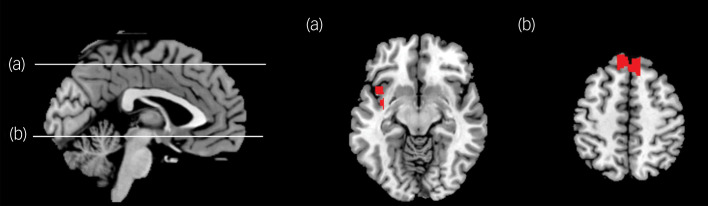
Region of interest (ROI)-based functional magnetic resonance imaging contrast analysis. Both sides of the medial prefrontal cortex, the anterior cingulate cortex, the amygdala and the insula were set as ROIs. Statistical inference was set as an uncorrected *P*-value height threshold of 0.001 in conjunction with an extent threshold correction of false-wise error rate of *P* < 0.05. (a) The interaction effects for the group-by-task condition were significant in the left insula. (b) The main effects of the group were significant in the frontal eye field.

In the task-based fMRI analyses, the interaction effects for the group × task condition were significant in the left insula (peak MNI coordinates: −36, −6, −10; cluster size *k* = 425; peak *z* = 4.56; *P* = 0.013, cluster-corrected; Fig. 2(a)). *Post hoc* analyses revealed that the PTSD group exhibited significantly stronger BOLD responses to interfering trauma-related stimuli in the left insula. The main effects of grouping (PTSD group and control group) were significant in the frontal eye field (FEF) (peak MNI coordinates: −10, 42, 52; *k* = 1007; peak *z* = 5.70; *P* < 0.001, cluster-corrected; Fig. 2(b)). *Post hoc* analyses revealed that the PTSD group had weaker BOLD activity in the FEF than the control group. In contrast, there was no significant cluster in which the main effects of the task were significant.

### Functional connectivity analysis in the left insula

**Fig. 3:**
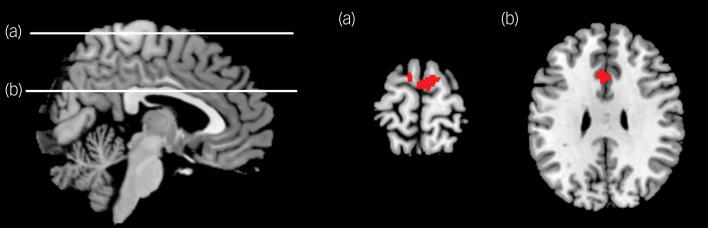
Left-insula-based functional connectivity analysis. The statistical inference was set as an uncorrected *P*-value height threshold of 0.001 in conjunction with an extent threshold correction of false-wise error rate of *P* < 0.05. (a) Compared with controls, participants with post-traumatic stress disorder (PTSD) showed significantly weaker functional connectivity between the left insula and the supplementary motor area. (b) Compared with controls, participants with PTSD showed significantly weaker functional connectivity between the left insula and the anterior cingulate cortex.

One left insula seed consisted of a 5 mm radius sphere centred on the coordinates *x* = −36, *y* = −6, *z* = 10, where the interaction effects were significant. The PTSD group had weaker insular functional connectivity with the supplementary motor area (peak MNI coordinates: 4, −4, 70; *k* = 258; peak *z* = 4.14; *P* = 0.007, cluster-corrected; Fig. 3(a)) and the ACC (peak MNI coordinates: −6, 20, 30; *k* = 189; peak *z* = 3.93; *P* = 0.031, cluster-corrected; Fig. 3(b)) than the control group. In contrast, there was no significant cluster in which the PTSD group had stronger insular functional connectivity than the controls.

### Correlation between functional connectivity and clinical variables

**Fig. 4:**
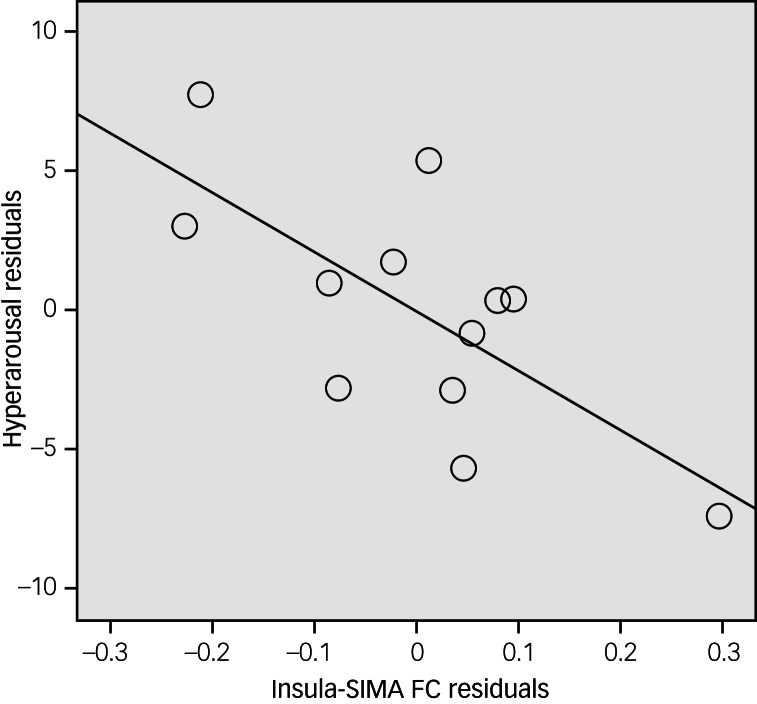
Partial correlation analyses after controlling for age and Beck Anxiety Inventory score. Non-standardised residuals were used to make scatter plots. Participants with PTSD exhibited a negative correlation between functional connectivity in the left insula–supplementary motor area and score on the Clinician-Administered PTSD Scale hyperarousal subscale (*r* = −0.699, *P* = 0.025).

A significant correlation was shown for functional connectivity between the left insula and the supplementary motor area such that the smaller the functional connectivity strength, the higher the CAPS hyperarousal subscale scores in the PTSD group (*r* = −0.699, *P* = 0.025; Fig. 4). Left insula–supplementary motor area connectivity strength was not significantly correlated with the other CAPS subscale scores (intrusion: *P* = 0.675; avoidance: *P* = 0.793). The correlation tests showed no statistical significance for functional connectivity between the left insula and ACC (intrusion: *P* = 0.641; avoidance: *P* = 0.553; hyperarousal: *P* = 0.710). There was no significant correlation between left insula–supplementary motor area connectivity and the CAPS hyperarousal subscale scores in the control group (*P* = 0.214).

## Discussion

### Insular activation

In this study, we evaluated brain activation in firefighters with and without PTSD during a behavioural Stroop match-to-sample task involving executive control. Participants with PTSD did not show any significant differences in accuracy of task completion compared with the controls. However, the PTSD group had longer reaction times when presented with trauma-related interference. This may reflect a stronger psychological response to trauma-related interferences in those with PTSD. Furthermore, the PTSD group showed greater functional activation of the left insular regions in response to interfering trauma-related stimuli while exercising executive control. We suggest that this insular hyperactivation may be related to the longer response times during exposure to trauma-related stimuli. However, to elucidate the link between the present findings and the clinical aspects of PTSD, further studies including more integrated behavioural assessments are needed.

The observed hyperactivation of the left insula in response to trauma-related interferences in the Stroop match-to-sample task might reflect hyperresponsiveness to trauma-related stimuli in the PTSD group. Our findings are consistent with those of previous studies that reported hyperactivation of the insular regions in PTSD during exposure to emotional stimuli.^26,27^ The insula is responsible for salience processing of emotional stimuli and linking it with cognitive control processing.^28^ We suggest that the PTSD group might experience trauma-related stimuli as more highly salient interferences while performing executive control. Although the insula is commonly examined in PTSD-related studies, the locations of brain clusters where functional alterations occur vary depending on the context in which traumatic stimuli are presented in task-based fMRI.^29^ For instance, previous studies on PTSD reported functional insular alterations on the right rather than the left in response to passively presented trauma-related scripts.^30,31^ The salience network in which the insula participates has been suggested to be bilateral.^32^ However, evidence suggests that the left insula is more prominently connected with the executive control network^33^ and correlated with behavioural adaptation for salience information.^34^ We consider that this study's presentation of trauma-related stimuli (i.e. inserted as interference during a Stroop task) is consistent with the findings of left-insular alterations.

### Functional connectivity

In functional connectivity analysis with the left insular seed region, the PTSD group exhibited weak functional connectivity of the left insula with the supplementary motor area and ACC. These findings coincide with a previous insula-based functional connectivity study of PTSD that reported weak functional connectivity between the left insula and the ACC.^35^ The ACC is associated with top-down executive functions^36^ involving conflict monitoring and decision-making. The supplementary motor area may also be involved in proper cognitive control.^37^ The weak insular functional connectivity in the supplementary motor area and the ACC observed in this study may reflect diminished cognitive control of salience processing in PTSD. Moreover, we showed that the lower functional connectivity between the left insula and the supplementary motor area was correlated with higher scores on PTSD symptom scales, particularly the CAPS hyperarousal subscale. Others have shown that impaired executive control contributes to dysfunctional coping strategies and other manifestations of PTSD^3^ and have suggested an inverse relationship between cognitive task performance and hyperarousal symptoms.^38^ Taken together, our findings suggest that the insular functional connectivity characteristics of firefighters with PTSD have a close relationship with their clinical features.

In addition to functional alterations in the insula, the PTSD group showed lower functional activity in the FEF than the controls in the task-based fMRI analysis. As one of the core brain regions of the dorsal attention network, the FEF may be involved in top-down attentional control,^39^ which plays an important role in the adaptive regulation of emotional responses in individuals with PTSD.^40^ The FEF is also involved in the modulation of eye movement and may be one of the neural correlates of eye-movement desensitisation and reprocessing therapy for PTSD.^41^ Despite a lack of significance in the group × task interaction effects, the group differences in BOLD activity in the FEF suggest that the FEF may contribute to the neurobiological pathophysiology of PTSD.

### Strengths and limitations

This study performed task-based fMRI to observe the differences in responses to interference during executive control tasks, not simply in response to trauma-related stimuli. Although many studies have examined the increased responsiveness of the insula to trauma-related stimuli, task-based fMRI studies exploring responses to implicitly inserted trauma-related interference are relatively scarce.^29^ Furthermore, this study was conducted for a special occupational group of firefighters who had not yet received psychiatric treatment.

However, this study has several limitations. First, the sample was too small to fully investigate functional brain characteristics of PTSD. The results of this study, which were not equally reproduced for all brain regions identified in previous studies, may be affected by this small sample size. Second, the homogeneity of the trauma experienced by participants in this study was not well controlled, and differences in trauma experiences may have affected the psychological responses to the photographic stimuli used in the study. Future studies that use larger samples of firefighters and control for types of trauma are needed to elaborate on the neuroimaging results of this study.

## Data Availability

The data that support the findings of this study are available from the corresponding author on reasonable request.
